# SECOND: Sparsely Embedded Convolutional Detection

**DOI:** 10.3390/s18103337

**Published:** 2018-10-06

**Authors:** Yan Yan, Yuxing Mao, Bo Li

**Affiliations:** 1 State Key Laboratory of Power Transmission Equipment and System Security and New Technology, Chongqing University, Chongqing 400044, China; scrin@foxmail.com; 2 TrunkTech Co., Ltd., No. 3, Danling street, ZhongGuan Town, HaiDian District, Beijing 100089, China; libo@trunk.tech

**Keywords:** 3D object detection, convolutional neural networks, LIDAR, autonomous driving

## Abstract

LiDAR-based or RGB-D-based object detection is used in numerous applications, ranging from autonomous driving to robot vision. Voxel-based 3D convolutional networks have been used for some time to enhance the retention of information when processing point cloud LiDAR data. However, problems remain, including a slow inference speed and low orientation estimation performance. We therefore investigate an improved sparse convolution method for such networks, which significantly increases the speed of both training and inference. We also introduce a new form of angle loss regression to improve the orientation estimation performance and a new data augmentation approach that can enhance the convergence speed and performance. The proposed network produces state-of-the-art results on the KITTI 3D object detection benchmarks while maintaining a fast inference speed.

## 1. Introduction

Great advances have been made in convolutional neural network (CNN)-based object detection [1,2], instance segmentation [3] and key-point detection [4] in recent years. This form of detection can be used in autonomous driving based on either monocular [5] or stereo images [6]. However, the methods used to process images cannot be applied directly to LiDAR data. This is a significant limitation for applications such as autonomous driving and robot vision. State-of-the-art methods can achieve an average precision (AP) of 90% of 2D car detection but only an AP of 15% [7] for 3D image-based car detection. To overcome the lack of spatial information provided by images alone, point cloud data have become increasingly important in 3D applications. Point cloud data contain accurate depth information and can be generated by LiDAR or RGB-D cameras.

Many current 3D detectors use a fusion method that exploits both images and point cloud data. Point cloud data are converted into a 2D bird’s eye view image [8] or are projected onto an image [9,10]. Features are then extracted using a convolutional network, and a fusion process is applied to map the features between the image and other views. In [11], the point cloud data are initially filtered using bounding boxes generated by a 2D detector, and a convolutional network is then used to directly process the points. In other methods, such as those of [12,13,14,15], the point cloud data are assigned to volumetric grid cells via quantization, and 3D CNNs are then applied.

Recently, a new approach called VoxelNet [14] has been developed. This approach combines raw point cloud feature extraction and voxel-based feature extraction in a single-stage end-to-end network. It first groups point cloud data into voxels and then applies linear networks voxel by voxel before converting the voxels into dense 3D tensors to be used in a region proposal network (RPN) [16]. At present, this is a state-of-the-art approach. However, its computational cost makes it difficult to use for real-time applications. In this paper, we present a novel approach called SECOND (**S**parsely **E**mbedded **CON**volutional **D**etection), which addresses these challenges in 3D convolution-based detection by maximizing the use of the rich 3D information present in point cloud data. This method incorporates several improvements to the existing convolutional network architecture. Spatially sparse convolutional networks are introduced for LiDAR-based detection and are used to extract information from the *z*-axis before the 3D data are downsampled to something akin to 2D image data. We also use a GPU (Graphics Processing Unit)-based rule generation algorithm for sparse convolution to increase the speed. In comparison to a dense convolution network, our sparse-convolution-based detector achieves a factor-of-4 speed enhancement during training on the KITTI dataset and a factor-of-3 improvement in the speed of inference. As a further test, we have designed a small model for real-time detection that has a run time of approximately 0.025 s on a GTX 1080 Ti GPU, with only a slight loss of performance.

Another advantage of using point cloud data is that it is very easy to scale, rotate and move objects by applying direct transformations to specified points on those objects. SECOND incorporates a novel form of data augmentation based on this capability. A ground-truth database is generated that contains the attributes of objects and the associated point cloud data. Objects sampled from this database are then introduced into the point clouds during training. This approach can greatly increase the convergence speed and the final performance of our network.

In addition to the above, we also introduce a novel angle loss regression approach to solve the problem of the large loss generated when the difference in orientation between the ground truth and the prediction is equal to π, which yields a bounding box identical to the true bounding box. The performance of this angle regression approach surpasses that of any current method we know about, including the orientation vector regression function available in AVOD [9]. We also introduce an auxiliary direction classifier to recognize the directions of objects.

At the time of submission, our method produces state-of-the-art results across all classes for KITTI-based 3D detection [7], while running at 20 fps for the larger model and 40 fps for the smaller one.

The key contributions of our work are as follows:

- We apply sparse convolution in LiDAR-based object detection, thereby greatly increasing the speeds of training and inference.
- We propose an improved method of sparse convolution that allows it to run faster.
- We propose a novel angle loss regression approach that demonstrates better orientation regression performance than other methods do.
- We introduce a novel data augmentation method for LiDAR-only learning problems that greatly increases the convergence speed and performance.

## 2. Related Work

Below, we briefly review existing works on 3D object detection based on point cloud data and images.

### 2.1. Front-View- and Image-Based Methods

Methods using 2D representations of RGB-D data can be divided into two classes: those based on a bird’s eye view (BEV) and those based on a front view. In typical image-based methods [5], 2D bounding boxes, class semantics and instance semantics are generated first, and then hand-crafted approaches are used to generate feature maps. Another method [17] uses a CNN to estimate 3D bounding boxes from images and a specially designed discrete-continuous CNN to estimate the orientations of objects. Methods using LiDAR [18] involve the conversion of point clouds into front-view 2D maps and the application of 2D detectors to localize objects in the front-view images. These methods have been shown to perform poorly for both BEV detection and 3D detection compared to other methods.

### 2.2. Bird’s-Eye-View-Based Methods

MV3D [8] is the first method to convert point cloud data into a BEV representation. In this method, point cloud data are converted into several slices to obtain height maps, and these height maps are then concatenated with the intensity map and density map to obtain multichannel features. ComplexYOLO [19] uses a YOLO (You Only Look Once) [20] network and a complex angle encoding approach to increase speed and orientation performance, but it uses fixed heights and *z*-locations in the predicted 3D bounding boxes. In [21], a fast single-stage proposal-free detector is designed that makes use of specific height-encoded BEV input. A key problem with all of these approaches, however, is that many data points are dropped when generating a BEV map, resulting in a considerable loss of information on the vertical axis. This information loss severely impacts the performance of these methods in 3D bounding box regression.

### 2.3. 3D-Based Methods

Most 3D-based methods either use point cloud data directly or require converting these data into 3D grids or voxels instead of generating BEV representations. In [12], point cloud data are converted into voxels containing feature vectors, and then a novel convolution-like voting-based algorithm is used for detection. Ref. [13] exploits sparsity in point cloud data by leveraging a feature-centric voting scheme to implement novel convolutions, thus increasing the computation speed. These methods use hand-crafted features, and while they yield satisfactory results on specific datasets, they cannot adapt to the complex environments commonly encountered in autonomous driving. In a distinct approach, the authors of [22,23] develop a system that could learn pointwise features directly from point clouds by means of a novel CNN-based architecture, whereas Ref. [24] uses a k-neighborhood method together with convolution to learn local spatial information from a point cloud. These methods directly process point cloud data to perform 1D convolution on k-neighborhood points, but they cannot be applied to a large number of points; thus, image detection results are needed to filter the original data points. Some CNN-based detectors convert point cloud data into voxels. In the method presented in [15], point cloud data are discretized into two-valued voxels, and then 3D convolution is applied. The method of [14] groups point cloud data into voxels, extracts voxelwise features, and then converts these features into a dense tensor to be processed using 3D and 2D convolutional networks. The major problem with these methods is the high computational cost of 3D CNNs. Unfortunately, the computational complexity of a 3D CNN grows cubically with the voxel resolution. In [25,26], a spatially sparse convolution is designed that increases the 3D convolution speed, whereas Ref. [27] proposes a new approach to 3D convolution in which the spatial structure of the output remains unchanged, which greatly increases the processing speed. In [28], submanifold convolution is applied for the 3D semantic segmentation task; however, there is no known method that uses sparse convolution for the detection task.

Similar to all of these approaches, our method makes use of a 3D convolutional architecture, but it incorporates several novel improvements.

### 2.4. Fusion-Based Methods

Some methods combine camera images with point clouds. For instance, the authors of [29] use a 3D RPN at two scales with different receptive fields to generate 3D proposals and then feed the 3D volume from the depth data of each 3D proposal into a 3D CNN and the corresponding 2D color patch into a 2D CNN to predict the final results. In the method presented in [8], point cloud data are converted into a front view and a BEV, and then the feature maps extracted from both point cloud maps are fused with an image feature map. The MV3D network with images performs better than the BEV-only network by a large margin, but this architecture does not work well for small objects and runs slowly because it contains three CNNs. The authors of [9] combine images with a BEV and then use a novel architecture to generate high-resolution features maps and 3D object proposals. In [11], 2D detection results are used to filter a point cloud such that PointNet [22] could then be applied to predict 3D bounding boxes. However, fusion-based methods typically run slowly because they need to process a significant amount of image input. The additional requirement of a time-synchronized and calibrated camera with LiDAR capabilities restricts the environments in which such methods can be used and reduces their robustness. Our method, by contrast, can achieve state-of-the-art performance using only LiDAR data.

## 3. SECOND Detector

In this section, we describe the architecture of the proposed SECOND detector and present the relevant details regarding training and inference.

### 3.1. Network Architecture

**Figure 1:**
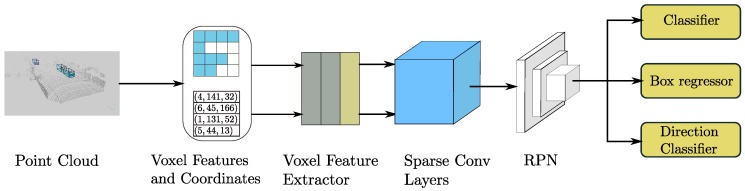
The structure of our proposed SECOND detector. The detector takes a raw point cloud as input, converts it to voxel features and coordinates, and applies two VFE (voxel feature encoding) layers and a linear layer. Then, a sparse CNN is applied. Finally, an RPN generates the detection.

The proposed SECOND detector, depicted in Figure 1, consists of three components: (1) a voxelwise feature extractor; (2) a sparse convolutional middle layer; and (3) an RPN.

#### 3.1.1. Point Cloud Grouping

Here, we follow the simple procedure described in [14] to obtain a voxel representation of point cloud data. We first preallocate buffers based on the specified limit on the number of voxels; then, we iterate over the point cloud and assign the points to their associated voxels, and we save the voxel coordinates and the number of points per voxel. We check the existence of the voxels based on a hash table during the iterative process. If the voxel related to a point does not yet exist, we set the corresponding value in the hash table; otherwise, we increment the number of voxels by one. The iterative process will stop once the number of voxels reaches the specified limit. Finally, we obtain all voxels, their coordinates and the number of points per voxel for the actual number of voxels. For the detection of cars and other objects in related classes, we crop the point cloud based on the ground-truth distribution at [−3,1]×[−40,40]×[0,70.4] m along the z×y×x axes. For pedestrian and cyclist detection, we use crop points at [−3,1]×[−20,20]×[0,48] m. For our smaller model, we use only points within the range of [−3,1]×[−32,32]×[0,52.8] m to increase the inference speed. The cropped areas need to be slightly adjusted based on the voxel size to ensure that the sizes of the generated feature maps can be correctly downsampled in the subsequent networks. For all tasks, we use a voxel size of $v_{D}=0.4\times v_{H}=0.2\times v_{W}=0.2$ m. The maximum number of points in each empty voxel for car detection is set to T=35, which is selected based on the distribution of the number of points per voxel in the KITTI dataset; the corresponding maximum for pedestrian and cyclist detection is set to T=45 because pedestrians and cyclists are relatively small and, consequently, more points are needed for voxelwise feature extraction.

#### 3.1.2. Voxelwise Feature Extractor

We use a voxel feature encoding (VFE) layer, as described in [14], to extract voxelwise features. A VFE layer takes all points in the same voxel as input and uses a fully connected network (FCN) consisting of a linear layer, a batch normalization (BatchNorm) layer and a rectified linear unit (ReLU) layer to extract pointwise features. Then, it uses elementwise max pooling to obtain the locally aggregated features for each voxel. Finally, it tiles the obtained features and concatenates these tiled features and the pointwise features together. We use $VFE(c_{out})$ to denote a VFE layer that transforms the input features into $c_{out}$-dimensional output features. Similarly, $FCN(c_{out})$ denotes a Linear-BatchNorm-ReLU layer that transforms the input features into $c_{out}$-dimensional output features. As a whole, the voxelwise feature extractor consists of several VFE layers and an FCN layer.

#### 3.1.3. Sparse Convolutional Middle Extractor

##### Review of Sparse Convolutional Networks

Ref. [25] was the first paper to introduce spatially sparse convolution. In this approach, output points are not computed if there is no related input point. This approach offers computational benefits in LiDAR-based detection because the grouping step for the point clouds in KITTI will generate 5k–8k voxels with a sparsity of nearly 0.005. As an alternative to normal sparse convolution, submanifold convolution [27] restricts an output location to be active if and only if the corresponding input location is active. This avoids the generation of too many active locations, which can lead to a decrease in speed in subsequent convolution layers due to the large number of active points.

##### Sparse Convolution Algorithm

Let us first consider the 2D dense convolution algorithm. We use $W_{u,v,l,m}$ to denote filtered elements and $D_{u,v,l}$ to denote image elements, where *u* and *v* are spatial location indices, *l* represents input channels and *m* represents output channels. The function P(x,y) generates the input locations that need to be computed given the provided output locations. Thus, the convolution output for $Y_{x,y,m}$ is given by the following formula:

(1) $$Y_{x,y,m}=\sum_{u,v\in P(x,y)}\sum_{l}W_{u-u_{0},v-v_{0},l,m}D_{u,v,l},$$

where *x* and *y* are the output spatial indexes and $u-u_{0}$ and $v-v_{0}$ represent the kernel-offset *u* and *v* coordinates. A general matrix multiplication (GEMM)-based algorithm (also known as the im2col-based algorithm [30]) can be used to gather all of the data needed to construct a matrix ${\tilde{D}}_{P(x,y),l}$ and then perform GEMM itself:

(2) $$Y_{x,y,m}=\sum_{l}W_{*,l,m}{\tilde{D}}_{P(x,y),l},$$

where $W_{*,l,m}$ corresponds to $W_{u-u_{0},v-v_{0},l,m}$ but in GEMM form. For the sparse data $D_{i,l}^{{}^{\prime }}$ and the associated output $Y_{j,m}^{{}^{\prime }}$, the direct calculation algorithm can be written as follows:

(3) $$Y_{j,m}^{{}^{\prime }}=\sum_{i\in P^{{}^{\prime }}\left(j\right)}\sum_{l}W_{k,l,m}D_{i,l}^{{}^{\prime }},$$

where $P^{{}^{\prime }}\left(j\right)$ is a function for obtaining the input index *i* and the filter offset. The subscript *k* is the 1D kernel offset that corresponds to $u-u_{0}$ and $v-v_{0}$ in Equation (1), and the subscript *i* corresponds to *u* and *v* in Equation (1). The GEMM-based version of Equation (3) is given by the following formula:

(4) $$Y_{j,m}^{{}^{\prime }}=\sum_{l}W_{*,l,m}{\tilde{D}}_{P^{{}^{\prime }}\left(j\right),l}^{{}^{\prime }}.$$

**Figure 2:**
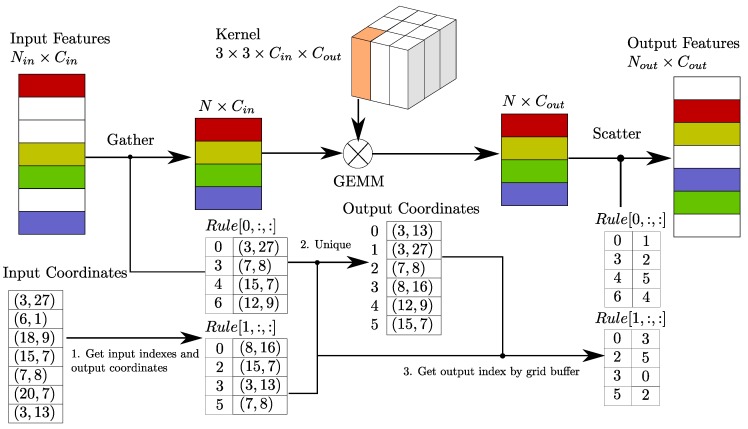
The sparse convolution algorithm is shown above, and the GPU rule generation algorithm is shown below. $N_{in}$ denotes the number of input features, and $N_{out}$ denotes the number of output features. *N* is the number of gathered features. Rule is the rule matrix, where Rule[i,:,:] is the *i*-th rule corresponding to the *i*-th kernel matrix in the convolution kernel. The boxes with colors except white indicate points with sparse data and the white boxes indicate empty points.

The gathered matrix ${\tilde{D}}_{P^{{}^{\prime }}\left(j\right),l}^{{}^{\prime }}$ of sparse data still contains many zeros that do not need to be computed. To solve this problem, instead of directly converting Equation (3) into Equation (4), we rewrite Equation (3) as follows:

(5) $$Y_{j,m}^{{}^{\prime }}=\sum_{k}\sum_{l}W_{k,l,m}{\tilde{D}}_{R_{k,j},k,l}^{{}^{\prime }},$$

where $R_{k,j}$, also called **Rule**, is a matrix that specifies the input index *i* given the kernel offset *k* and the output index *j*. The inner sum in Equation (5) cannot be calculated via GEMM, so we need to gather the necessary input to construct the matrix, perform GEMM, and then scatter the data back. In practice, we can gather the data directly from the original sparse data by using a preconstructed input–output index rule matrix. This increases the speed. In detail, we construct a rule matrix table $R_{k,i,t}=R\left[k,i,t\right]$ with dimensions of $K\times N_{in}\times 2$, where *K* is the kernel size (expressed as a volume), $N_{in}$ is the number of input features and *t* is the input/output index. The elements R[:,:,0] store the input indexes for gathering, and the elements R[:,:,1] store the output indexes for scattering. The top part of Figure 2 shows our proposed algorithm.

##### Rule Generation Algorithm

The major performance challenges confronting current implementations [31] are associated with the rule generation algorithm. A CPU-based rule generation algorithm using a hash table is typically used, but such an algorithm is slow and requires data transfer between the CPU and GPU. A more direct approach to rule generation is to iterate over the input points to find the outputs related to each input point and store the corresponding indexes into the rules. During the iterative process, a table is needed to check the existence of each output location to decide whether to accumulate the data using a global output index counter. This is the greatest challenge hindering the use of parallel computing in the algorithm.

In our case, we have designed a GPU-based rule generation algorithm (Algorithm 1) that runs faster on a GPU. The bottom part of Figure 1 shows our proposed algorithm. First, we collect the input indexes and associated spatial indexes instead of the output indexes (1st loop in Algorithm 1). Duplicate output locations are obtained in this stage. We then execute a unique parallel algorithm on the spatial index data to obtain the output indexes and their associated spatial indexes. A buffer with the same spatial dimensions as those of the sparse data is generated from the previous results for table lookup in the next step (2nd loop in Algorithm 1). Finally, we iterate on the rules and use the stored spatial indexes to obtain the output index for each input index (3rd loop in Algorithm 1). Table 1 shows a performance comparison between our implementation and existing approaches.
**Algorithm 1: 3D Rule Generation**
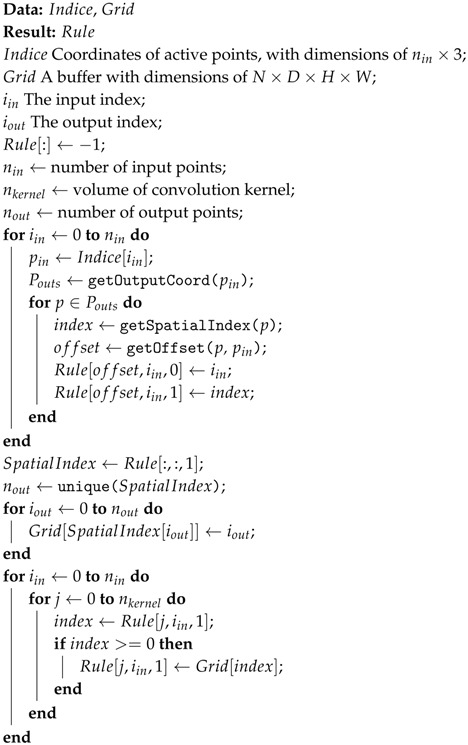


##### Sparse Convolutional Middle Extractor

**Figure 3:**
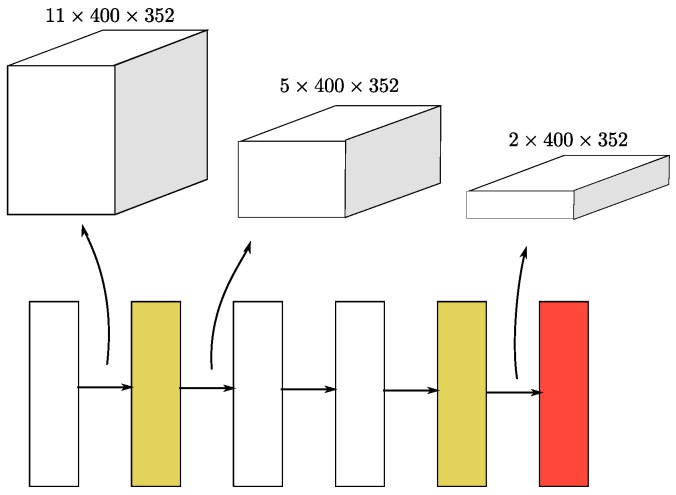
The structure of our proposed sparse middle feature extractor. The yellow boxes represent sparse convolution, the white boxes represent submanifold convolution, and the red box represents the sparse-to-dense layer. The upper part of the figure shows the spatial dimensions of the sparse data.

Our middle extractor is used to learn information about the *z*-axis and convert the sparse 3D data into a 2D BEV image. Figure 3 shows the structure of the middle extractor. It consists of two phases of sparse convolution. Each phase contains several submanifold convolutional layers and one normal sparse convolution to perform downsampling in the *z*-axis. After the *z*-dimensionality has been downsampled to one or two, the sparse data are converted into dense feature maps. Then, the data are simply reshaped into image-like 2D data.

#### 3.1.4. Region Proposal Network

RPNs [1] have recently begun to be used in many detection frameworks. In this work, we use a single shot multibox detector (SSD)-like [32] architecture to construct an RPN architecture. The input to the RPN consists of the feature maps from the sparse convolutional middle extractor. The RPN architecture is composed of three stages. Each stage starts with a downsampled convolutional layer, which is followed by several convolutional layers. After each convolutional layer, BatchNorm and ReLU layers are applied. We then upsample the output of each stage to a feature map of the same size and concatenate these feature maps into one feature map. Finally, three 1 × 1 convolutions are applied for the prediction of class, regression offsets and direction.

#### 3.1.5. Anchors and Targets

Because the objects to be detected are of approximately fixed sizes, we use fixed-size anchors determined based on the means of the sizes and center locations of all ground truths in the KITTI training set with rotations of 0 and 90 degrees. For cars, we use an anchor with dimensions of w=1.6×l=3.9×h=1.56 m, centered at z=−1.0 m. For pedestrians, we use an anchor with dimensions of w=0.6×l=0.8×h=1.73 m, and for cyclists, the anchor has dimensions of w=0.6×l=1.76×h=1.73 m; both are centered at z=−0.6 m.

Each anchor is assigned a one-hot vector of classification targets, a 7-vector of box regression targets and a one-hot vector of direction classification targets. Different classes have different thresholds for matching and nonmatching. For cars, the anchors are assigned to ground-truth objects using an intersection-over-union (IoU) threshold of 0.6 and are assigned to the background (negative) if their IoUs are less than 0.45. Anchors with IoUs between 0.45 and 0.6 are ignored during training. For pedestrians and cyclists, we use values of 0.35 for the nonmatching threshold and 0.5 for the matching threshold.

For the regression targets, we use the following box encoding functions:

(6) $$\begin{array}{c} x_{t}=\frac{x_{g}-x_{a}}{d_{a}},y_{t}=\frac{y_{g}-y_{a}}{d_{a}},z_{t}=\frac{z_{g}-z_{a}}{h_{a}},\\ w_{t}=\log\left(\frac{w_{g}}{w_{a}}\right),l_{t}=\log\left(\frac{l_{g}}{l_{a}}\right),h_{t}=\log\left(\frac{h_{g}}{h_{a}}\right),\\ \theta_{t}=\theta_{g}-\theta_{a}, \end{array}$$

where *x*, *y*, and *z* are the center coordinates; *w*, *l*, and *h* are the width, length, and height, respectively; θ is the yaw rotation around the *z*-axis; the subscripts *t*, *a*, and *g* indicate the encoded value, the anchor, and the ground truth, respectively; and $d^{a}=\sqrt{{\left(l^{a}\right)}^{2}+{\left(w^{a}\right)}^{2}}$ is the diagonal of the base of the anchor box.

### 3.2. Training and Inference

#### 3.2.1. Loss

##### Sine-Error Loss for Angle Regression

Previous methods of angle regression, including corner encoding, direct encoding and vector encoding, usually exhibit poor performance. The corner prediction approach [8] cannot determine the direction of an object and cannot be used for pedestrian detection, for which the BEV boxes are nearly square. The vector encoding approach [9,19] retains redundant information and leads to difficulty detecting far-away objects based on LiDAR. VoxelNet [14] directly predicts the radian offset but is subject to an adversarial example problem between the cases of 0 and π radians because these two angles correspond to the same box but generate a large loss when one is misidentified as the other. Our architecture solves this problem by introducing a new angle loss regression:

(7) $$L_{\theta }=\mathrm{SmoothL}1\left(\sin\left(\theta_{p}-\theta_{t}\right)\right),$$

where the subscript *p* indicates the predicted value. This approach to angle loss has two advantages: (1) it solves the adversarial example problem between orientations of 0 and π, and (2) it naturally models the IoU against the angle offset function. To address the issue that this loss treats boxes with opposite directions as being the same, we have added a simple direction classifier to the output of the RPN. This direction classifier uses a softmax loss function. We use the following approach to generate the direction classifier target: if the yaw rotation around the *z*-axis of the ground truth is higher than zero, the result is positive; otherwise, it is negative.

##### Focal Loss for Classification

Our network usually generates ∼70k anchors within a KITTI point cloud. Unfortunately, there are usually only a few ground truths, each of which generates only 4–6 positives. This leads to an extreme imbalance between the foreground and background classes. The authors of RetinaNet [33] introduced an effective single-stage loss, called the focal loss, that can solve this problem; therefore, we use this loss in our architecture. The classification loss has the following form:

(8) $$FL\left(p_{t}\right)=-\alpha_{t}{\left(1-p_{t}\right)}^{\gamma }\log\left(p_{t}\right),$$

where $p_{t}$ is the model’s estimated probability and α and γ are the parameters of the focal loss. We use α=0.25 and γ=2 in our training process.

##### Total Training Loss

By combining the losses discussed above, we can obtain the final form of the multitask loss as follows:

(9) $$L_{total}=\beta_{1}L_{cls}+\beta_{2}\left(L_{reg-\theta }+L_{reg-other}\right)+\beta_{3}L_{dir},$$

where $L_{cls}$ is the classification loss, $L_{reg-other}$ is the regression loss for location and dimension, $L_{reg-\theta }$ is our novel angle loss, and $L_{dir}$ is the direction classification loss. $\beta_{1}=1.0$, $\beta_{2}=2.0$, and $\beta_{3}=0.2$ are constant coefficients of our loss formula. We use a relatively small value of $\beta_{3}$ to avoid cases in which our network would struggle to recognize the directions of objects.

#### 3.2.2. Data Augmentation

##### Sample Ground Truths from the Database

The major problem we encountered during training was the existence of too few ground truths, which significantly limited the convergence speed and final performance of the network. To solve this problem, we introduced a data augmentation approach. First, we generated a database containing the labels of all ground truths and their associated point cloud data (points inside the 3D bounding boxes of the ground truths) from the training dataset. Then, during training, we randomly selected several ground truths from this database and introduced them into the current training point cloud via concatenation. Using this approach, we could greatly increase the number of ground truths per point cloud and simulate objects existing in different environments. To avoid physically impossible outcomes, we performed a collision test after sampling the ground truths and removed any sampled objects that collided with other objects.

##### Object Noise

To consider noise, we followed the same approach used in VoxelNet [14], in which each ground truth and its point cloud are independently and randomly transformed, instead of transforming all point clouds with the same parameters. Specifically, we used random rotations sampled from a uniform distribution Δθ∈[−π/2,π/2] and random linear transformations sampled from a Gaussian distribution with a mean of zero and a standard deviation of 1.0.

##### Global Rotation and Scaling

We applied global scaling and rotation to the whole point cloud and to all ground-truth boxes. The scaling noise was drawn from the uniform distribution [0.95,1.05], and [−π/4,π/4] was used for the global rotation noise.

#### 3.2.3. Optimization

The proposed SECOND detector was trained using stochastic gradient descent (SGD). We used an Adam optimizer run on a GTX 1080 Ti GPU with a total of three point clouds per minibatch. All models were trained for 160 epochs (200k iterations). The initial learning rate was 0.0002, with an exponential decay factor of 0.8 and a decay every 15 epochs. A decay weight of 0.0001, a beta1 value of 0.9 and a beta2 value of 0.999 were used. Training the large car detection network with a single GTX 1080 Ti GPU took 19 h, and only 9 h was needed to train the smaller model.

#### 3.2.4. Network Details

We propose the use of two networks: a large one and a small one. Points that lie outside the camera view frustum need to be removed.

**Figure 4:**
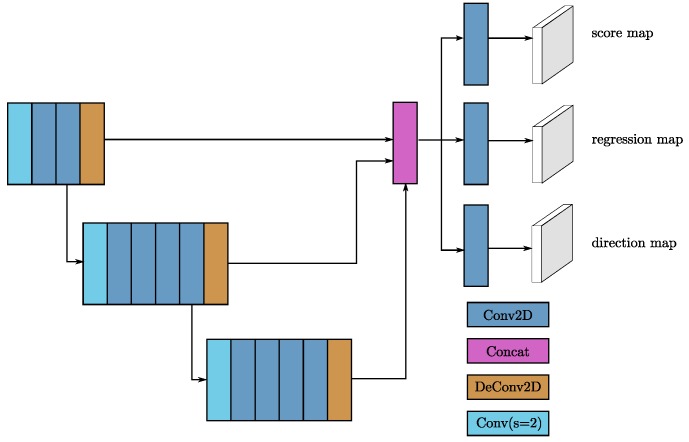
The detailed structure of the RPN. Blue boxes represent convolutional layers, purple boxes represent layers for concatenation, sky blue boxes represent stride-2 downsampling convolutional layers, and brown boxes represent transpose convolutional layers.

For car detection, two VFE layers are used in SECOND, namely, VFE(32) and VFE(128) for the large network and VFE(32) and VFE(64) for the smaller network, following a Linear(128) layer. Thus, the dimensions of the output sparse tensor are 128×10×400×352 for the large network and 128×10×320×264 for the small network. Then, we use a two-stage sparse CNN for feature extraction and dimension reduction, as shown in Figure 3. Each convolutional layer follows a BatchNorm layer and a ReLU layer. All sparse convolutional layers have a 64-output feature map, a kernel size of (3,1,1) kernel size and a stride of (2,1,1). The dimensions of the output of the middle block are 64×2×400×352 for the large network. Once the output has been reshaped to 128×400×352, the RPN network can be applied. Figure 4 shows the architecture of the RPN. We use $Conv2D(c_{out},k,s)$ to represent a Conv2D-BatchNorm-ReLU layer and $DeConv2D(c_{out},k,s)$ to represent a DeConv2D-BatchNorm-ReLU layer, where $c_{out}$ is the number of output channels, **k** is the kernel size and **s** is the stride. Because all layers have the same size across all dimensions, we use scalar values for **k** and **s**. All Conv2D layers have the same padding, and all DeConv2D layers have zero padding. In the first stage of our RPN, three Conv2D(128,3,1(2)) layers are applied. Then, five Conv2D(128,3,1(2)) layers and five Conv2D(256,3,1(2)) layers are applied in the second and third stages, respectively. In each stage, s=2 only for the first convolutional layer; otherwise, s=1. We apply a single DeConv2D(128,3,s) layer for the last convolution in each stage, with s=1, 2, and 4 for the three stages, sequentially. For pedestrian and cyclist detection, the only difference with respect to car detection is that the stride of the first convolutional layer in the RPN is 1 instead of 2.

## 4. Experiments

We trained our network on the KITTI dataset [34] and evaluated our 3D object detector on the KITTI benchmarks [7] for 3D object detection and BEV object detection, which include Car, Pedestrian and Cyclist benchmarks. For each class, the detector was evaluated for three levels of difficulty: easy, moderate and hard. The difficulty assessment was based on the object height in the 2D results, occlusion and truncation. We followed the approach proposed in [8] by splitting the provided 7481 training examples into a training set of 3712 samples and an evaluation set of 3769 samples. Because of limited access to the test server, we evaluated our larger model using only the test set and thus can provide an assessment of performance on the validation set only for our smaller model. The BEV and 3D detection results were evaluated in terms of the AP. We compared our proposed method with several state-of-the art methods. Here, we focus on the car detection results because pedestrian and cyclist detection may need images to get better results, as described in Section 4.3. Our computation environment for inference included a Core-i5 6500 CPU (4 cores), 16 GB of DDR4 memory and a GTX 1080 Ti GPU.

### 4.1. Evaluation Using the KITTI Test Set

Table 2 presents the performance of our 3D detector on the KITTI test set. Our method achieves state-of-the-art results with only LiDAR data and is superior to the original VoxelNet [14] by a significant margin, while AVOD-FPN [9] uses both image and LiDAR data and uses a custom 85/15 training/validation split (as opposed to our 50/50 split) and ground plane estimation to improve the results. F-PointNet [11] uses a 2D detector that has been fine-tuned using ImageNet weights, whereas our network is trained from scratch and uses only LiDAR data. For pedestrian and cyclist detection, our results are comparable to the state-of-the-art results. Table 3 shows the performance of our method for 3D BEV object localization. Our method performs slightly worse than the state-of-the-art methods but still achieves comparable results, and it performs better than the LiDAR-only VoxelNet [14] network.

**Figure 5:**
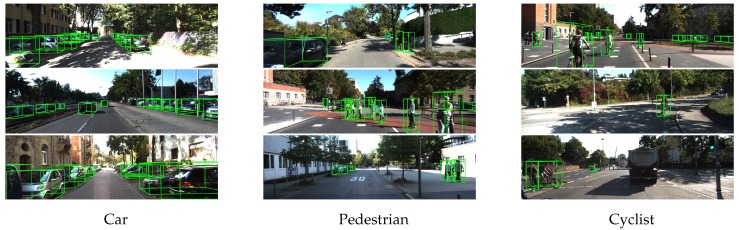
Results of 3D detection on the KITTI test set. For better visualization, the 3D boxes detected using LiDAR are projected onto images from the left camera.

We present several 3D detection results in Figure 5. The 3D bounding boxes have been projected into the camera coordinate system. Overall, this evaluation shows that our network can produce high-accuracy results with a fast inference speed.

### 4.2. Evaluation Using the KITTI Validation Set

As no currently published method except VoxelNet [14] presents validation results for pedestrian and cyclist detection, we show only our validation results for car detection for comparison with other methods.

We report the performance achieved on the KITTI validation set in Table 4 and Table 5. Both our large and small networks outperform all the competing approaches across all levels of difficulty, while our small network maintains a particularly fast inference speed.

### 4.3. Analysis of the Detection Results

**Figure 6:**
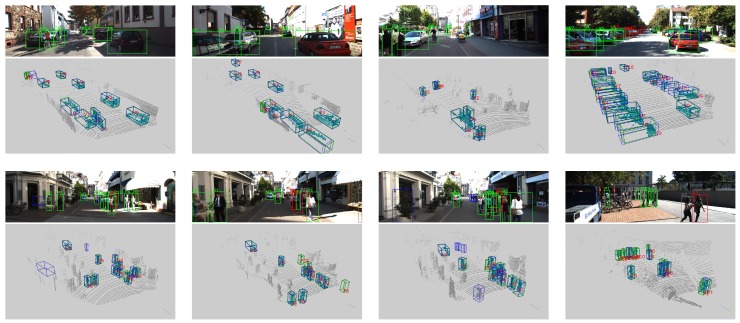
Results of detection on the KITTI validation set. In each image, a green box indicates successful detection, a red box indicates detection with low accuracy, a gray box indicates a false negative, and a blue box indicates a false positive. The digit and letter beside each box represent the instance ID and the class, respectively, with “V” denoting a car, “P” denoting a pedestrian and “C” denoting a cyclist. In the point clouds, green boxes indicate ground truths, and blue boxes indicate detection results.

Figure 6 shows some detection results for point clouds and related images.

#### 4.3.1. Car Detection

The first four images and the related point clouds in Figure 6 are shown as examples of typical car detection. Our network can produce excellent results for cars at moderate and close distances. For cars that are farther away, our network still produces good results despite the few available points, which make it very challenging to detect such objects in an image. Moreover, our network can surprisingly detect strongly overlapping cars, for which the point clouds contain only a small proportion of the original car point clouds. Generally, these results demonstrate the effectiveness of the 3D aspects of our network.

On the other hand, some major failures in car detection, including inaccurate rotation and size estimation, can be observed for cases with only a few points in the point cloud. Our network missed many cars that were farther away from the LiDAR, typically containing fewer than 10 points. There is also a noticeable lack of labels: some far-away or heavily occluded cars are not labeled at all, although the network did successfully detect these vehicles.

#### 4.3.2. Pedestrian and Cyclist Detection

The last four images and the related point clouds in Figure 6 show detection results for pedestrians and cyclists. Here, there are more false positives and false negatives than there were for cars. Some false positives present on unreasonable locations in the image. This difficulty can be attributed to the typically higher instance densities of pedestrians and cyclists compared with that of cars, with fewer points per instance, which cause pedestrians and cyclists to be more easily confused with other point clusters and noise. In addition, the relatively small volumes of pedestrians and cyclists lead to less voxels of them, which limits the power of the CNNs. However, the promising aspect of this finding is that if it is possible to use information from such images, unrelated points can simply be filtered and locations of objects can be easily determined based on 2D detection results, which should make this problem easy to eliminate.

### 4.4. Ablation Studies

#### 4.4.1. Sparse Convolution Performance

Table 1 shows the performance of our improved sparse convolution method and compares it with that of the original implementation in SparseConvNet [31]. It can be seen that our sparse convolution approach is faster than the original implementation because of its faster rule generation.

#### 4.4.2. Different Angle Encodings

Table 6 shows the performances of different angle encoding methods on the KITTI validation set. It can be seen that our method of handling angles performs better than the angle vector encoding used in AVOD [9] and ComplexYOLO [19]. In our method, the direction classification weight can be controlled to make the network focus more on overlap maximization instead of struggling with direction recognition in difficult cases.

#### 4.4.3. Sampling Ground Truths for Faster Convergence

**Figure 7:**
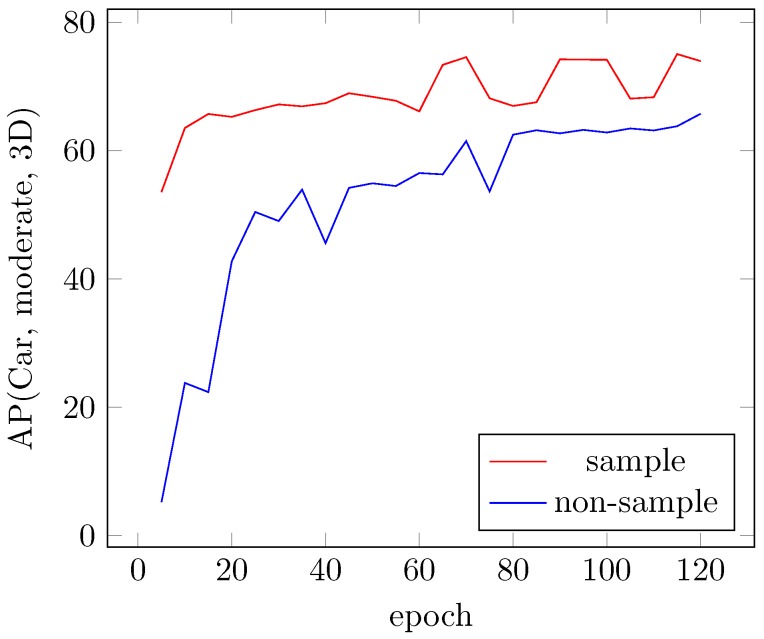
Sampling vs. nonsampling methods for 3D map evaluation on the KITTI validation set (Car class, moderate difficulty).

To rectify the extreme data imbalance between the positive and negative examples during training, we introduced a technique for sampling ground truths and their associated point cloud data to construct better training data on the fly. Figure 7 shows the performance curves for training with and without ground-truth sampling on the KITTI validation set for the Car class. This figure shows that our sampling approach greatly increases the convergence speed and enhances the final results.

## 5. Conclusions

Most existing methods for 3D object detection convert point cloud data into 2D representations, such as BEV and front-view representations, and thus lose most of the spatial information contained in the original point clouds. In this paper, we have introduced a novel method of angle loss regression, have successfully applied sparse convolution in a LiDAR-based network and have proposed a novel approach to data augmentation that makes full use of the advantages of point clouds. Experiments on the KITTI dataset have shown that the proposed network outperforms other state-of-the-art approaches. Additionally, the proposed architecture has been shown to run in real time. However, our network shows lower performance for pedestrian and cyclist detection and for BEV detection. Future work will include the investigation of methods for joint camera- and LiDAR-based detection, such as the fusion of image features with LiDAR voxel features, to enhance the detection performance and the use of weakly supervised training to exploit the large unlabeled portion of the KITTI dataset.

## Figures and Tables

**Table 1:** Comparison of the execution speeds of various convolution implementations. SparseConvNet is the official implementation of submanifold convolution [27]. All benchmarks were run on a GTX 1080 Ti GPU with the data from the KITTI dataset.

**Sparse Convolution (1 layer)**			
**Channels**	**SECOND**	**SpConvNet [31]**	**Dense**
64×64	8.6	21.2	567
128×128	13.8	24.8	1250
256×256	25.3	37.4	N/A
512×512	58.7	86.0	N/A
**Submanifold Convolution (4 layers)**			
**Channels**	**SECOND**	**SpConvNet [31]**	**Dense**
64×64	7.1	16.0	N/A
128×128	11.3	21.5	N/A
256×256	20.4	37.0	N/A
512×512	49.0	94.1	N/A

**Table 2:** 3D detection performance: Average precision (AP) (in %) for 3D boxes in the KITTI test set. In AVOD and AVOD-FPN [9], a custom 85/15 training/validation split and ground plane estimation are adopted to improve the results. For F-PointNet [11], a GTX 1080 GPU, which has 67% of the peak performance of a GTX 1080 Ti (used for our method) or a Titan Xp (used for AVOD), was used for inference. The bold number indicates the best result in a table.

Method	Time (s)	Car			Pedestrian			Cyclist		
		Easy	Moderate	Hard	Easy	Moderate	Hard	Easy	Moderate	Hard
MV3D [8]	0.36	71.09	62.35	55.12	N/A	N/A	N/A	N/A	N/A	N/A
MV3D (LiDAR) [8]	0.24	66.77	52.73	51.31	N/A	N/A	N/A	N/A	N/A	N/A
F-PointNet [11]	0.17	81.20	70.39	62.19	**51.21**	**44.89**	**40.23**	**71.96**	**56.77**	**50.39**
AVOD [9]	0.08	73.59	65.78	58.38	38.28	31.51	26.98	60.11	44.90	38.80
AVOD-FPN [9]	0.1	81.94	71.88	**66.38**	46.35	39.00	36.58	59.97	46.12	42.36
VoxelNet (LiDAR) [14]	0.23	77.47	65.11	57.73	39.48	33.69	31.51	61.22	48.36	44.37
SECOND	**0.05**	**83.13**	**73.66**	66.20	51.07	42.56	37.29	70.51	53.85	46.90

**Table 3:** Bird’s eye view detection performance: Average precision (AP) (in %) for BEV boxes in the KITTI test set.

Method	Time (s)	Car			Pedestrian			Cyclist		
		Easy	Moderate	Hard	Easy	Moderate	Hard	Easy	Moderate	Hard
MV3D [8]	0.36	86.02	76.90	68.49	N/A	N/A	N/A	N/A	N/A	N/A
MV3D (LiDAR) [8]	0.24	85.82	77.00	68.94	N/A	N/A	N/A	N/A	N/A	N/A
F-PointNet [11]	0.17	88.70	84.00	75.33	**58.09**	**50.22**	**47.20**	**75.38**	**61.96**	**54.68**
AVOD [9]	0.08	86.80	**85.44**	77.73	42.51	35.24	33.97	63.66	47.74	46.55
AVOD-FPN [9]	0.1	88.53	83.79	77.90	50.66	44.75	40.83	62.39	52.02	47.87
VoxelNet (LiDAR) [14]	0.23	**89.35**	79.26	77.39	46.13	40.74	38.11	66.70	54.76	50.55
SECOND	**0.05**	88.07	79.37	**77.95**	55.10	46.27	44.76	73.67	56.04	48.78

**Table 4:** 3D detection performance: Average precision (AP) (in %) for 3D boxes in the KITTI validation set.

Method	Time (s)	Easy	Moderate	Hard
MV3D [8]	0.36	71.29	62.68	56.56
F-PointNet [11]	0.17	83.76	70.92	63.65
AVOD-FPN [9]	0.1	84.41	74.44	68.65
VoxelNet [14]	0.23	81.97	65.46	62.85
SECOND	0.05	**87.43**	**76.48**	**69.10**
SECOND (small)	**0.025**	85.50	75.04	68.78

**Table 5:** Bird’s eye view detection performance: Average precision (AP) (in %) for BEV boxes in the KITTI validation set.

Method	Time (s)	Easy	Moderate	Hard
MV3D [8]	0.36	86.55	78.10	76.67
F-PointNet [11]	0.17	88.16	84.02	76.44
VoxelNet [14]	0.23	89.60	84.81	78.57
SECOND	0.05	**89.96**	**87.07**	**79.66**
SECOND (small)	**0.025**	89.79	86.20	79.55

**Table 6:** A comparison of the performances of different angle encoding methods on the KITTI validation set for the Car class.

Method	Easy	Moderate	Hard
Vector [9]	85.99	74.79	67.82
SECOND	**87.43**	**76.48**	**69.10**
